# Cytosolic and ER-associated ribosomes share rRNA 2′-O-methylation landscapes across human cell types

**DOI:** 10.1261/rna.081054.126

**Published:** 2026-08

**Authors:** Ülkü Uzun, Anders H. Lund

**Affiliations:** Biotech Research and Innovation Centre, Faculty of Health and Medical Sciences, University of Copenhagen, 2200 Copenhagen, Denmark

**Keywords:** ribosome heterogeneity, rRNA 2′-O-methylation, RiboMeth-seq, local translation, neuronal translation

## Abstract

Ribosome heterogeneity arising from variable rRNA 2′-O-methylation (2′-O-Me) has been proposed as a potential mechanism for translational specialization, but whether such heterogeneity contributes to compartment-specific translation remains unknown. Here, we systematically compare the 2′-O-Me landscapes of cytosolic and endoplasmic reticulum (ER)–associated ribosomes across three human cell types: HEK293 cells, H9-derived neural progenitor cells (NPCs), and neurons differentiated from these NPCs. Using detergent-based fractionation combined with RiboMeth-seq, we generate site-resolved rRNA methylation profiles for each compartment. Within each cell type, cytosolic and ER-associated ribosomes display highly similar 2′-O-Me patterns, with only modest compartment-specific differences observed at 18S:462 in NPCs and 28S:2043 in neurons. Across all samples, differences in 2′-O-Me patterns are more pronounced between cell types than between compartments. Together, these findings indicate that 2′-O-Me does not establish a broad ER-specific methylation signature, and is unlikely to be a major determinant of ribosome localization or function at the ER.

## INTRODUCTION

Ribosomes, long viewed as uniform molecular machines, are now recognized as dynamic regulators of translation with the capacity for functional specialization (Aspden et al. 2025). Such ribosomal heterogeneity can arise through multiple mechanisms, including rRNA sequence variation, incorporation of ribosomal protein (RP) paralogs, altered RP stoichiometry, and extensive rRNA modifications (Genuth and Barna 2018). Among these features, 2′-O-methylation (2′-O-Me) represents the most abundant rRNA modification in human cells. It is catalyzed by the methyltransferase fibrillarin in complex with C/D box snoRNAs (SNORDs) that provide site specificity (Sloan et al. 2017; Gay et al. 2022). Notably, many 2′-O-Me sites are only partially modified in the ribosome pool, generating ribosome subpopulations with distinct modification states that differ across cell types, developmental contexts, and disease states (Krogh et al. 2016; Marcel et al. 2020).

A growing body of work demonstrates that variation in rRNA 2′-O-Me can influence translation, cell identity, and cellular behavior. For example, methylation at 18S:174 modulates decoding of GC- and AU-rich codons, shaping proliferation and metabolic programs; loss of 28S:3904 biases human stem cells toward neuroectoderm by altering translation of developmental regulators; decreased methylation at 28S:2402 contributes to ZEB1-driven epithelial–mesenchymal transition and a claudin-low breast cancer phenotype; and reduced methylation at 18S:1447 impairs leukemia stem-cell maintenance, extending survival in mouse models (Jansson et al. 2021; Häfner et al. 2023; Zhou et al. 2023; Morin et al. 2025). Together, these findings establish rRNA methylation heterogeneity as a key layer of translational control that shapes cell-fate decisions. However, whether such heterogeneity also contributes to compartment-specific translation within cells remains an open question.

Given the potential for compositional variation among ribosomes, an important question is how molecular heterogeneity integrates with the spatial organization of translation in eukaryotic cells (Bourke et al. 2023). Protein synthesis is compartmentalized within distinct subcellular domains to coordinate local physiological demands, such as those occurring near mitochondria (Luo et al. 2025), the nuclear envelope (Yang et al. 2025), or synaptic terminals (Holt et al. 2019). Spatial regulation ensures precise timing and dosage of protein synthesis, supports proper folding and posttranslational modification, and safeguards proteostasis by limiting the accumulation of mistargeted or aggregation-prone intermediates. A major site of spatially organized translation is the endoplasmic reticulum (ER). Although ER-associated ribosomes can serve as a global translation platform, proteins destined for secretion, membrane insertion, or organellar localization are predominantly synthesized on ER-associated ribosomes (Chen et al. 2011). Ribosome association with the ER can occur through at least three nonexclusive mechanisms: (i) cotranslational targeting mediated by signal peptides or transmembrane domains recognized by the signal recognition particle (SRP), (ii) persistent ribosome–ER interactions enabling re-initiation cycles on membrane-associated ribosomes, and (iii) mRNA-dependent localization to the ER, independent of SRP or ongoing translation, potentially through interactions with RNA-binding proteins or sequence elements within untranslated regions (Sánchez et al. 2025). Together, these pathways imply that ribosomes with distinct molecular features, including specific rRNA modifications, may be preferentially recruited to or stabilized at the ER, leading to compositional differences between ER-associated and cytosolic ribosome subpopulations.

Accordingly, cytosolic and ER-associated ribosomes coexist as partially distinct translational populations with well-documented functional differences. The ER provides a specialized environment for cotranslational protein folding, N-glycosylation, and disulfide-bond formation, while minimizing aggregation of hydrophobic proteins and reducing cytosolic stress (Braakman and Hebert 2013). Translation on the ER has been reported to enhance protein yield compared with cytosolic translation in certain mammalian systems, underscoring its importance for the synthesis of long or highly structured proteins (Stephens and Nicchitta 2008; Reid and Nicchitta 2012; Horste et al. 2023). Previous work has primarily focused on how rRNA 2′-O-Me heterogeneity shapes translation across cell types, developmental stages, and disease states, but it remains unknown whether this heterogeneity is spatially partitioned across subcellular translation sites.

**FIGURE 1.**
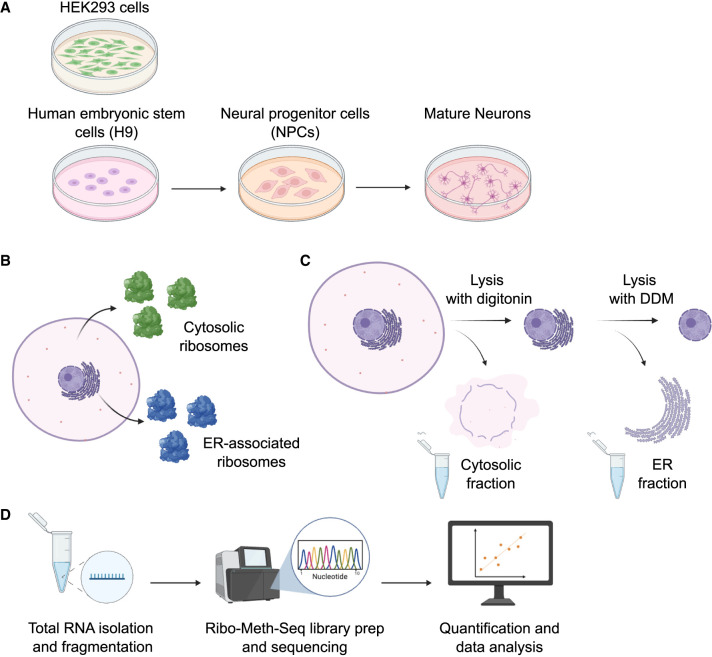
Experimental overview of cell systems, subcellular fractionation, and rRNA 2′-O-methylation profiling. (*A*) Schematic of the cellular models used in this study: HEK293 cells as well as human embryonic stem cells (H9) differentiated into neural progenitor cells (NPCs) and mature neurons. (*B*) Overview of the experimental comparison between cytosolic and ER-associated ribosomes for rRNA 2′-O-methylation profiling. (*C*) Detergent-based subcellular fractionation strategy used to isolate cytosolic and ER compartments. Cells are first permeabilized with digitonin to release cytosolic contents, yielding the cytosolic fraction, followed by solubilization of ER membranes with *n*-dodecyl-β-d-maltoside (DDM) to obtain the ER fraction while leaving nuclei intact. (*D*) Workflow for RiboMeth-seq analysis of rRNA 2′-O-methylation: total RNA isolation from cytosolic and ER fractions, RNA fragmentation, RiboMeth-seq library preparation and sequencing, and subsequent quantification and computational analysis of site-specific 2′-O-methylation levels.

Here, we asked whether cytosolic and ER-associated ribosomes differ in their 2′-O-Me landscapes, and whether such differences might contribute to compartment-specific translation. To address this, we profiled 2′-O-Me patterns of ribosomes isolated from cytosolic and ER-enriched fractions across three human cell types—HEK293 cells, neural progenitor cells (NPCs), and neurons. Using detergent-based fractionation combined with RiboMeth-seq, we obtained high-quality, site-resolved methylation profiles from each compartment. This data set allowed us to assess both cell-type–specific methylation heterogeneity and potential compartment-specific differences (Fig. 1).

Our results reveal that although rRNA 2′-O-Me patterns differ markedly between cell types, ER-associated and cytosolic ribosomes within the same cell type share highly similar methylation profiles, with only subtle compartment-specific differences in NPCs and neurons.

## RESULTS

### Isolation of cytosolic and ER-associated ribosomes

To test whether ER-associated ribosomes differ in their 2′-O-Me landscapes from cytosolic ribosomes, we used a detergent-based subcellular fractionation protocol to isolate cytosolic and ER compartments (Fig. 1B,C). Cells were first permeabilized with 0.015% digitonin to release cytosolic contents while preserving ER membranes, followed by a mild 0.004% digitonin wash to minimize cross-contamination. Digitonin was chosen for its ability to maintain translocon integrity and preserve the in vitro translation capacity of ER-associated ribosomes, as demonstrated by electron microscopy and functional studies (Voorhees and Hegde 2016; Pauwels et al. 2023). The ER fraction was then solubilized with 2% *n*-dodecyl-β-d-maltoside (DDM), leaving nuclei intact (Child et al. 2021). rRNA purified from cytosolic and ER fractions was subjected to RiboMeth-seq to quantify site-specific 2′-O-Me across ribosomal RNAs (Birkedal et al. 2015). Across all three cell types, the ER fraction consistently accounted for ∼22% of total A260 absorbance, indicating a reproducible and substantial contribution of ER-associated rRNA to the total ribosome pool (Supplemental Fig. S1). RiboMeth-seq profiles were highly reproducible across biological replicates in all conditions (Supplemental Fig. S2).

### ER-associated and cytosolic ribosomes share 2′-O-Me patterns in HEK293 cells

**FIGURE 2.**
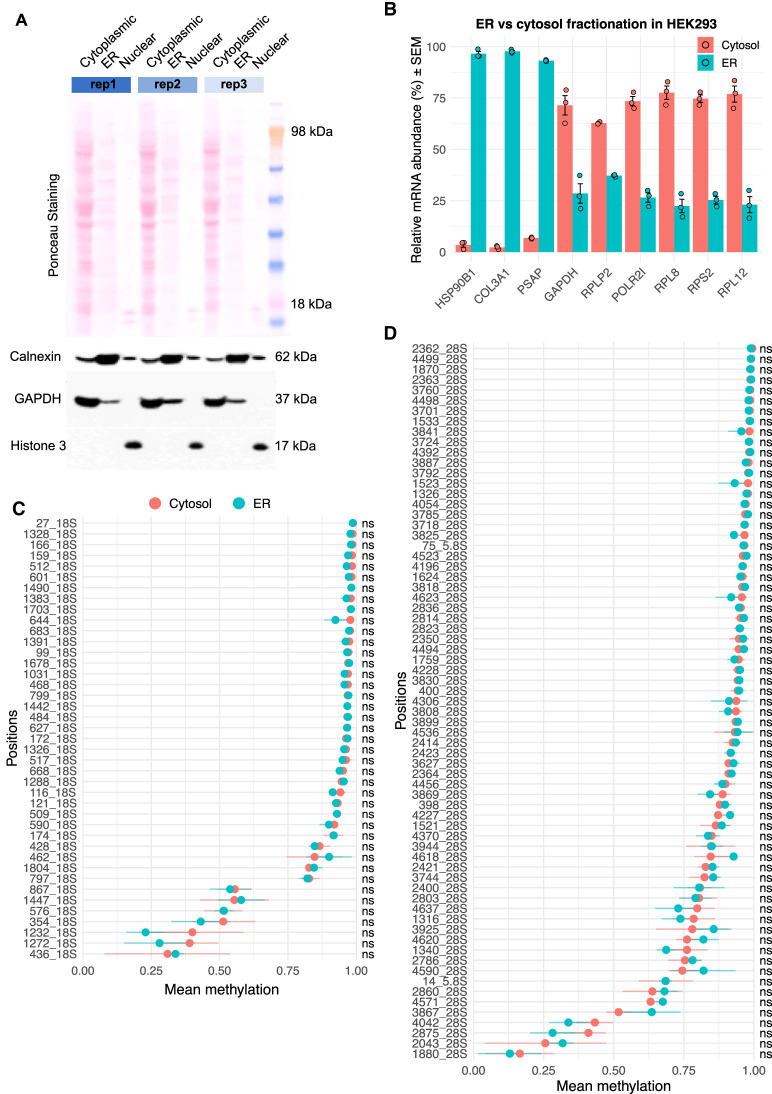
ER versus cytosolic 2′-O-methylation profiles of ribosomes in HEK293 cells. (*A*) Detergent-based fractionation of HEK293 cells into cytoplasmic, ER, and nuclear compartments, assessed by Ponceau staining and immunoblotting for the ER marker Calnexin (62 kDa), the cytosolic marker GAPDH (37 kDa), and the nuclear marker Histone 3 (17 kDa). Three independent biological replicates (rep1–rep3) are shown. (*B*) Relative mRNA distribution of the ER-enriched transcripts *HSP90B1*, *PSAP*, *COL3A1*, and the cytosolic transcripts *GAPDH*, *POLR2I*, *RPL8*, *RPLP2*, *RPS2*, and *RPL12* between cytosolic and ER fractions, calculated as percentage of total signal per gene. Data represent three biological replicates. (*C*) Mean 2′-O-methylation levels of small subunit (18S) rRNA sites in cytosolic and ER-associated ribosomes from HEK293 cells, quantified by RiboMeth-seq. Sites are ordered from highest to lowest mean methylation across all samples. (*D*) Mean 2′-O-methylation levels of large subunit (28S and 5.8S) rRNA sites in cytosolic and ER-associated ribosomes from HEK293 cells, quantified by RiboMeth-seq. Sites are ordered from highest to lowest mean methylation across all samples. In *C* and *D*, points represent the mean of three biological replicates per compartment, and error bars indicate the standard deviation. (ns) No significant difference between ER and cytosolic fractions for the indicated site (two-sided *t*-test, *P* ≥ 0.05 and absolute methylation difference ≤0.15). 28S rRNA positions are numbered according to the human NCBI RefSeq sequence NR_003287.4 (see Materials and Methods; Supplemental Table S1 for details).

We initially focused on HEK293 cells, which exhibit robust ER and secretory pathway activity (Malm et al. 2022). Successful separation of cytosolic and ER compartments was verified by enrichment of the cytoplasmic marker GAPDH and the ER marker Calnexin, as well as by qPCR for cytosolic- and ER-enriched mRNAs (Fig. 2A,B; Supplemental Fig. S3A). Western blot analysis indicated high purity of both cytosolic and ER fractions, while transcript analysis confirmed strong enrichment of ER-targeted (∼95%) and cytosolic (∼75%) transcripts (Fig. 2A,B; Supplemental Fig. S3A).

RiboMeth-seq analysis of known 2′-O-Me sites revealed no significant differences in methylation levels between rRNA from ER-associated and cytosolic ribosomes in HEK293 cells (Fig. 2C,D; Supplemental Fig. S3B,C). As expected, approximately two-thirds of sites were fully methylated, whereas the remaining sites displayed fractional methylation, indicating the presence of heterogeneous ribosome subpopulations. These observations suggest that, in HEK293 cells, the same heterogeneous pool of 2′-O-methylated ribosomes engages in translation both in the cytosol and at the ER. Consistently, fractionated RiboMeth-seq profiles closely resembled those obtained from HEK293 cells polysomes (Supplemental Fig. S3D).

### ER-associated and cytosolic ribosomes in neural progenitor cells show largely similar 2′-O-Me profiles

**FIGURE 3.**
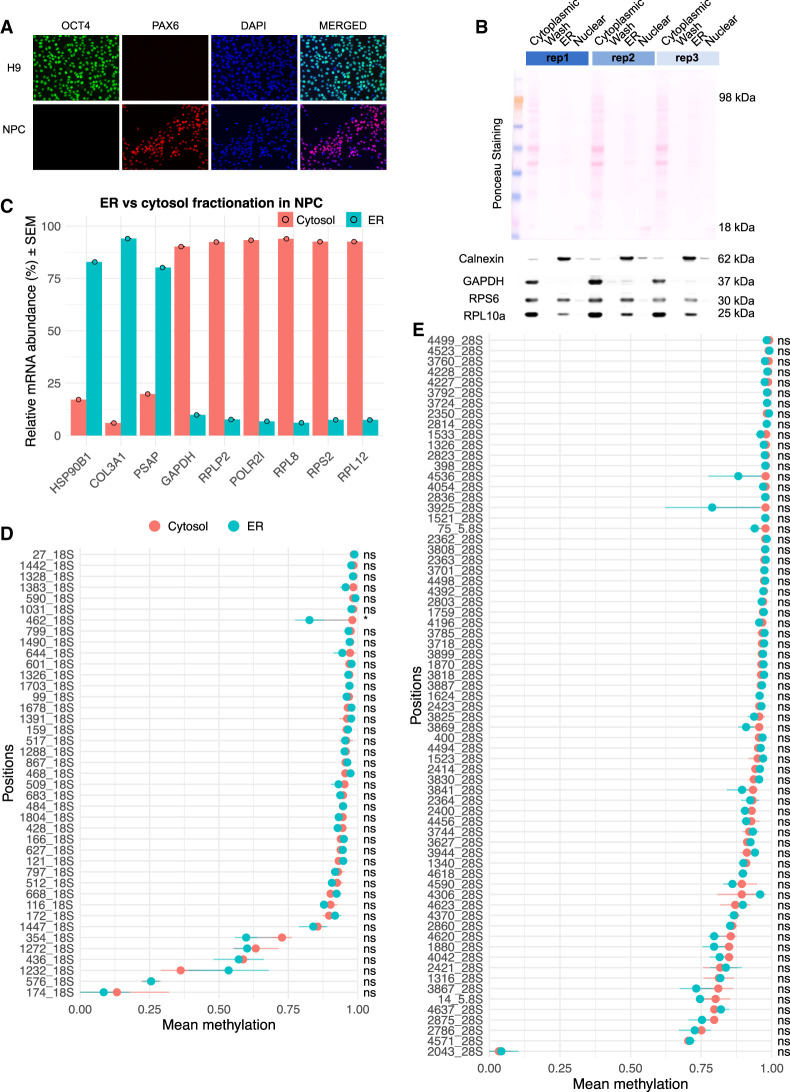
Validation of ER–cytosol fractionation and rRNA methylation profiles in human NPCs. (*A*) Immunofluorescence staining of pluripotent H9 cells (OCT4, DAPI) and derived neural progenitor cells (NPCs; PAX6, DAPI) confirms efficient neural induction. (*B*) Ponceau staining of cytoplasmic, wash, ER, and nuclear fractions from three independent NPC preparations demonstrates reproducible protein recovery across fractions, and western blot analysis of Calnexin and GAPDH verifies enrichment of ER and cytosolic markers at the expected molecular weights. RPS6 and RPL10a shows presence of small and large ribosomal proteins, respectively. (*C*) RT-qPCR of *HSP90B1*, *PSAP*, *COL3A1*, *GAPDH*, *POLR2I*, *RPL8*, *RPLP2*, *RPS2*, and *RPL12* mRNAs shows distinct relative distributions between cytosolic and ER fractions in NPCs, indicating successful compartmental separation. Data represent two technical replicates. (*D*) Mean 2′-O-methylation levels of small subunit (18S) rRNA (*E*) large subunit (28S and 5.8S) rRNA sites in cytosolic and ER-associated ribosomes in NPCs, quantified by RiboMeth-seq. Sites are ordered from highest to lowest mean methylation across all samples. Points represent the mean of three biological replicates per compartment, and error bars indicate the standard deviation. (ns) No significant difference between ER and cytosolic fractions for the indicated site (two-sided *t*-test, *P* ≥ 0.05 and absolute methylation difference ≤0.15). (*) Significant difference (*P* < 0.05). For 28S rRNA numbering, see Materials and Methods and Supplemental Table S1.

We next examined NPCs, in which translational control contributes to lineage specification (Blair et al. 2017). NPCs were differentiated from H9 human embryonic stem cells, and successful differentiation was confirmed by the expression of the NPC marker PAX6 (Fig. 3A). Application of the fractionation protocol to NPCs again yielded well-separated cytosolic and ER fractions, as assessed by protein markers and qPCR, confirming strong enrichment of ER-targeted (∼95%) and cytosolic (∼80%) transcripts (Fig. 3B,C; Supplemental Fig. S4A).

In this context, RiboMeth-seq profiles of ER-associated and cytosolic ribosomes were highly similar (Fig. 3D,E; Supplemental Fig. S4B,C). Among all analyzed sites, 18S:462 showed a modest but statistically significant compartment-specific difference, with methylation levels changing from 98% in cytosolic ribosomes to 83% in ER-associated ribosomes (*P* = 0.049), whereas no other positions exhibited significant differences in 2′-O-Me between compartments (Fig. 3D; Supplemental Fig. S5A,B; Khatter et al. 2015). Thus, rRNA 2′-O-Me heterogeneity appears to be largely shared between ER-associated and cytosolic ribosomes in NPCs.

### Neuronal ER-associated ribosomes display a modest 2′-O-Me shift at 28S:2043

**FIGURE 4.**
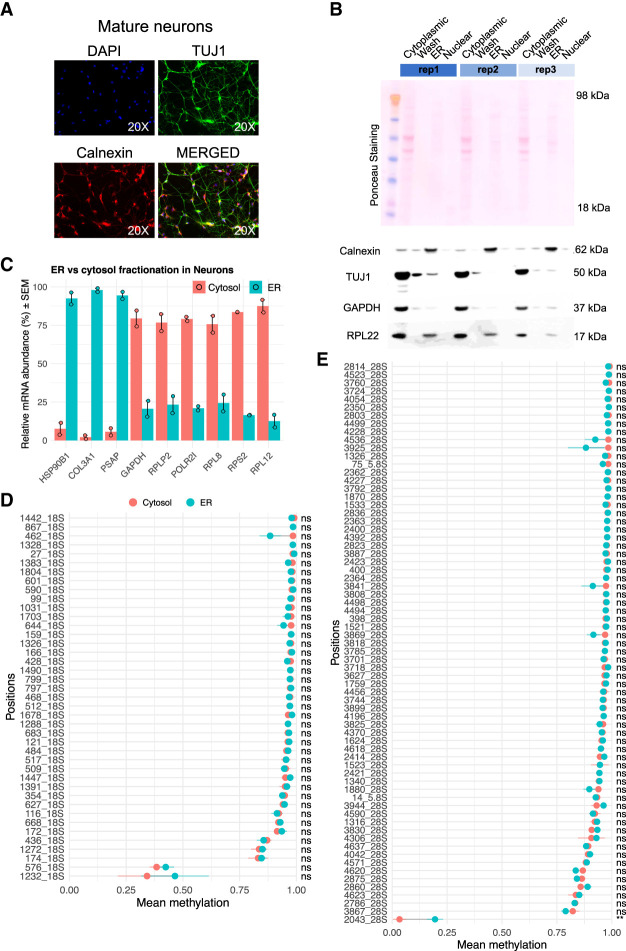
Validation of ER–cytosol fractionation and rRNA methylation profiles in mature neurons. (*A*) Immunofluorescence staining of mature neurons (DAPI, TUJ1, Calnexin) shows neuronal identity and ER localization in soma and neurites. (*B*) Detergent-based fractionation of neuronal cultures into cytoplasmic, wash, ER, and nuclear compartments, assessed by Ponceau staining and immunoblotting for Calnexin (62 kDa), TUJ1 (50 kDa), GAPDH (37 kDa), and RPL22 (17 kDa) across three biological replicates (rep1–rep3). (*C*) Relative mRNA distribution of the ER-enriched transcripts *HSP90B1*, *PSAP*, *COL3A1* and the cytosolic transcripts *GAPDH*, *POLR2I*, *RPL8*, *RPLP2*, *RPS2*, and *RPL12* between cytosolic and ER fractions, calculated as percentage of total signal per gene. Data represent two biological replicates. (*D*) Mean 2′-O-methylation levels of small subunit (18S) rRNA sites in cytosolic and ER-associated ribosomes from neurons, quantified by RiboMeth-seq. Sites are ordered from highest to lowest mean methylation across all samples. (*E*) Mean 2′-O-methylation levels of large subunit (28S and 5.8S) rRNA sites in cytosolic and ER-associated ribosomes from neurons, quantified by RiboMeth-seq. Sites are ordered from highest to lowest mean methylation across all samples. In *D* and *E*, points represent the mean of three biological replicates per compartment, and error bars indicate the standard deviation. (ns) No significant difference between ER and cytosolic fractions for the indicated site (two-sided *t*-test, *P* ≥ 0.05 and absolute methylation difference ≤0.15). (**) Significant difference (*P* < 0.01). For 28S rRNA numbering, see Materials and Methods and Supplemental Table S1.

Because neurons rely extensively on compartmentalized translation, particularly at distal processes, we next analyzed H9-derived neurons (Holt et al. 2019). Neuronal identity was validated by morphology and expression of neuronal marker TUJ1 (Fig. 4A). As in the other cell types, the fractionation protocol produced distinct cytosolic and ER fractions, confirmed by protein markers and transcript analysis. Western blot analysis indicated high purity for both fractions, with qPCR confirming strong enrichment of ER-targeted (∼95%) and cytosolic (∼80%) transcripts (Fig. 4B,C; Supplemental Fig. S6A).

Globally, RiboMeth-seq revealed that 2′-O-Me patterns were largely shared between ER-associated and cytosolic ribosomes in neurons (Fig. 4D,E; Supplemental Fig. S6C,D). Consistent with previous observations (Häfner et al. 2023), neurons generally exhibit higher overall 2′-O-Me levels (Fig. 4D,E; Supplemental Fig. S2E,F). Higher overall 2′-O-Me levels were also observed for more differentiated tissues compared to cancer cells and may be attributable to differences in the proportion of proliferating versus resting cells (Krogh et al. 2020). This could explain why maturating (non-dividing) neurons gradually accumulate higher levels of 2′-O-Me. Within this largely stable methylation landscape, a single site—28S:2043—displayed a compartment-specific difference in methylation, with 19% of ER-associated ribosomes methylated at this position compared with ∼3% of cytosolic ribosomes (*P* = 0.009; Fig. 4E; Supplemental Figs. S6B, S7A; Khatter et al. 2015). Because RiboMeth-seq scores at lowly methylated positions can be affected by 5′ and 3′ read counts at neighboring nucleotides, we examined normalized end-read profiles in a ±10 nt window around 28S:2043 and observed similar local coverage in cytosolic and ER fractions (Supplemental Fig. S7B), indicating that the observed difference is not driven by local read artifacts but reflects a genuine compartment-specific change in 2′-O-Me. This neuron-specific site represents a modest compartment-associated divergence within an otherwise similar 2′-O-Me profile.

### Cell type and compartment patterns in rRNA 2′-O-Me

**FIGURE 5.**
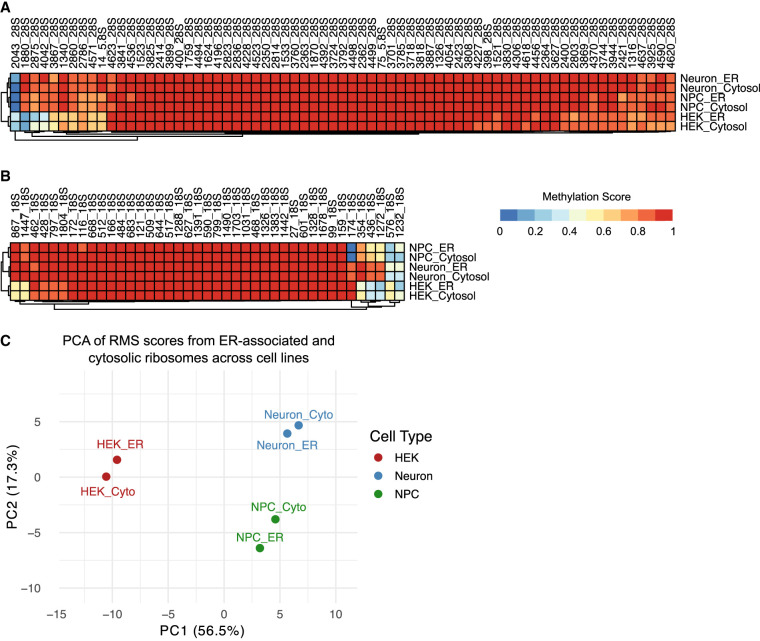
ER-associated and cytosolic ribosome rRNA 2′-O-methylation profiles across cell types. (*A*) Heat map of averaged signal intensities for 41 small subunit (18S) rRNA 2′-O-methylation sites across six experimental conditions (HEK cytosol, HEK ER, NPC cytosol, NPC ER, neuron cytosol, neuron ER). Rows correspond to individual rRNA positions and columns to cell type and subcellular localization; for each condition, values represent the mean of three replicates, hierarchically clustered by similarity, with color indicating relative magnitude from low (blue) to high (red). (*B*) Heat map of averaged signal intensities for 69 large subunit (28S and 5.8S) rRNA 2′-O-methylation sites across the same six conditions, displayed and clustered as in *A*. For 28S rRNA numbering, see Materials and Methods and Supplemental Table S1. (*C*) Principal component analysis (PCA) of RiboMeth-seq scores from ER-associated and cytosolic ribosomes in HEK293 cells, human NPCs, and neurons, showing that samples cluster primarily by cell type rather than subcellular compartment. Each point represents an individual sample; PC1 and PC2 explain 56.5% and 17.3% of the variance, respectively.

rRNA 2′-O-Me profiles were next compared across all fractions and cell types using hierarchical clustering and principal component analysis (PCA) of RiboMeth-seq data. Hierarchical clustering visualized as heat maps showed that samples segregate primarily by cell type, with clear separation of HEK293, NPC, and neuronal ribosomes (Fig. 5A,B), indicating pronounced cell type–specific 2′-O-methylation patterns and heterogeneity. Methylation sites such as 18S:174 and 28S:1880 exhibited more dynamic changes across the different cell types. Consistent with earlier analyses, 60%–70% of all methylation sites were highly methylated regardless of cell type. PCA likewise revealed clustering by cell type (Fig. 5C). In contrast, ER-associated and cytosolic fractions from the same cell type clustered closely together in both the heat maps and PCA, underscoring the overall similarity of their 2′-O-Me landscapes. Taken together, these findings suggest that rRNA 2′-O-Me patterns are largely shared between cytosolic and ER-associated ribosomes across different cell lines, arguing against a central role for this heterogeneity in directing ribosome localization or function at the ER, with only subtle compartment-specific changes at 18S:462 in NPCs and 28S:2043 in neurons.

## DISCUSSION

rRNA 2′-O-Me has emerged as an important source of ribosome heterogeneity with the potential to modulate translation in context-specific ways. Motivated by this concept, we asked whether differences in rRNA 2′-O-Me might contribute to spatial specialization of translation at the ER. Across the three human cell types examined, HEK293 cells, NPCs, and neurons, we did not identify a distinct 2′-O-Me signature that defines a generic ER-associated ribosome. Instead, ER-associated and cytosolic ribosomes displayed highly similar modification profiles, with only modest site-specific differences detected in NPCs and neurons.

In NPCs, a small difference was observed at 18S:462, where nearly all cytosolic ribosomes were methylated (98%) compared with 83% of ER-associated ribosomes. Although subtle, this reduction hints at a minor subpopulation of ER-associated ribosomes lacking methylation at a site located in the small ribosomal subunit, which participates in mRNA selection and decoding. Such variation could influence the translation of specific mRNA subsets within this compartment. To our knowledge, this position has not previously been reported as dynamically regulated across cellular conditions.

In neurons, we observed a compartment-specific difference at 28S:2043, which was methylated in 19% of ER-associated ribosomes but only 3% of cytosolic ribosomes. This site lies within the large subunit and has proximity to regions implicated in ribosome–translocon interactions (Gemmer et al. 2023), raising the possibility that local structural effects could subtly influence ER-associated translation. Notably, this site has also been reported to decrease upon MYC overexpression in BJ^hTERT^ cells (Jansson et al. 2021), although its functional relevance in neurons remains unclear.

The magnitude of these methylation differences, when considered relative to the total cellular ribosome pool, is small. Because ∼22% of ribosomes localize to the ER, a 15%–16% change in methylation within this fraction represents only ∼3%–4% of total ribosomes. Bulk RiboMeth-seq is limited in resolving such minor subpopulations and may partially reflect cell-to-cell heterogeneity rather than robust compartment-specific remodeling. Nevertheless, even a 3% subpopulation represents thousands of ribosomes per cell and could meaningfully impact translation of a restricted set of transcripts.

Our analysis compared cytosolic and ER-enriched ribosomes, but ribosome organization at the ER is likely far more complex. ER–ribosome interactions arise through several mechanisms—including cotranslational targeting via the signal recognition particle, mRNA-dependent localization, and persistent membrane association. Each may engage distinct ribosome pools. Furthermore, structural studies have further identified at least four ER translocon complexes that interact with ribosomes depending on the class of nascent protein (Gemmer et al. 2023). Thus, rather than a simple cytosol-versus-ER methylation code, a more intricate landscape of ribosome subpopulations may exist that is masked by bulk fractionation.

Importantly, we observed partially methylated ribosomes in both compartments, supporting the presence of heterogeneous ribosome populations overall. This heterogeneity may indicate that 2′-O-Me does not serve as a primary regulator of ER-associated translation, or alternatively, that its regulatory functions operate within smaller, highly specialized ribosome subsets that cannot be resolved with current methods.

A limitation of this study is that we rely on a detergent-based fractionation strategy. Digitonin permeabilization has been widely used to separate cytosolic and ER compartments while preserving nontranslating ribosomes on the ER and ribosome–translocon interactions, as shown by functional and cryo-EM studies (Schaletzky and Rapoport 2006; Voorhees and Hegde 2016; Pauwels et al. 2023). Consistent with this, we observe strong enrichment of ER markers (∼90%–95%) and clear segregation of cytosolic transcripts, indicating robust fraction purity across cell types.

However, a subset of cytosolic mRNA markers was also detected in ER fractions. This distribution is consistent with biological co-localization, as previous studies have shown that transcripts encoding cytosolic proteins can associate with and translated by ER-associated ribosomes (Stephens and Nicchitta 2008; Hoffman et al. 2019). In contrast, a previous study using ultracentrifugation-based fractionation reported sharper separation of these markers (Supplemental Fig. S8; Villanueva et al. 2024), possibly because ribosomes translating cytosolic mRNAs on the ER are less stably associated and more prone to dissociation during centrifugation. While minor technical carryover of cytosolic ribosomes in the ER fractions cannot be fully excluded, such contributions would likely be insufficient to mask substantial 2′-O-Me differences. Distinguishing true biological overlap from residual contamination will therefore require orthogonal purification strategies or higher-resolution approaches, such as proximity labeling or single-ribosome methods, in future studies.

Together, our findings argue that large-scale spatial compartmentalization of translation at the ER is unlikely to result from major differences in rRNA 2′-O-Me. However, they do not exclude the existence of functionally distinct ribosome subpopulations within either the cytosol or the ER. Other rRNA modifications, ribosome-associated proteins, assembly intermediates, or the local biochemical environment—including tRNA availability, translation factor composition, and mRNA targeting mechanisms—may confer specificity to ER-associated translation (Reid and Nicchitta 2015). Future work integrating single-ribosome or single-cell measurements with targeted perturbation of individual modification sites will be necessary to determine how minor ribosome subpopulations contribute to ER-localized translation in both physiological and disease contexts.

## MATERIALS AND METHODS

### Cell lines

HEK293 cells were maintained in DMEM (Gibco 31966-047); cells were passaged approximately two times a week using Trypsin-EDTA (0.25%) (Gibco 25200056). H9 human embryonic stem cell (hESC) lines (female) were maintained on plates coated with Matrigel (Corning Life Sciences 354277) in mTeSR Plus medium (Stem Cell Technologies 100-0276). The culture medium was changed every other day. Cells were passaged every three days using 1× TrypLE Select (Life Technologies 12563-011) reaching 80%–90% confluence in a medium supplemented with Rock inhibitor (Y-27632) (LC Laboratories Y-5301) at a final concentration of 10 µM. For cryopreservation, cells were frozen in a solution of 50% mTeSR Plus medium, 40% FBS 10% DMSO.

### Neuronal differentiation

H9 cells were differentiated into neural progenitor cells (NPCs) using the STEMdiff SMADi Neural Induction Medium (STEMCELL Technologies 08581), following the monolayer protocol, according to the manufacturer's instructions. Briefly, H9 cells were plated on Matrigel-coated plates at a density of 2 × 10^6^ cells/cm^2^ in neural induction medium supplemented with 10 µM ROCK inhibitor. The medium was changed daily, and the cells were passaged after 7 days. Following two additional passages (over the course of 2 wk) in neural induction medium, the cells were transitioned to STEMdiff Neural Progenitor Medium (STEMCELL Technologies 05833) for expansion, cryopreservation, or further differentiation.

H9-derived NPCs were further differentiated into forebrain neurons following the manufacturer's instructions. First, NPCs were plated at a density of 125,000 cells/cm^2^ on Matrigel-coated plates in STEMdiff Forebrain Neuron Differentiation Medium (STEMCELL Technologies 08600) for 1 wk with daily medium changes. The cells were then dissociated using Accutase (STEMCELL Technologies 07920) and replated at a density of 4–6 × 10^4^ cells/cm^2^ in STEMdiff Forebrain Neuron Maturation Medium (STEMCELL Technologies 08605) on plates coated with poly-l-ornithine (Sigma-Aldrich P3655; 15 µg/mL in PBS) and laminin (Sigma-Aldrich L2020; 5 µg/mL in DMEM/F-12). The medium was changed every other day, and the neurons were matured in culture for 22 days.

### Chemical fractionation of the cytosol and ER compartments

For HEK293 cells, 7 × 10^6^ cells were seeded per 15 cm dish 2 days before harvest. NPCs were seeded at 7 × 10^6^ cells per 10 cm dish 2 days before harvest, whereas neurons were plated at 1.8 × 10^6^ cells per 10 cm dish and differentiated for 22 days before collection. On the day of collection, cells were washed once with prewarmed growth medium (DMEM or DMEM/F12, as appropriate) and detached using Accutase for NPCs, trypsin for neurons, or by direct scraping for HEK293 cells. Cells were pelleted at 200*g* for 3 min at room temperature, resuspended in PBS lacking Ca^2+^ and Mg^2+^, and centrifuged again at 2000*g* for 1 min at 4°C, after which supernatants were removed completely.

Lysis and wash buffers were prepared with digitonin or DDM in the common base buffer (20 mM Tris-HCl pH 7.4, 150 mM NaCl, 5 mM MgCl_2_, 1 mM DTT, EDTA-free protease inhibitor cocktail [Roche, 11873580001], murine RNase inhibitor [NEB, M0314], and cycloheximide [100 µg/mL final; Sigma-Aldrich, C7698]) as described below. Digitonin stock was prepared at 2% (w/v) in DMSO (Sigma-Aldrich, D141) and stored at −20°C for up to 1 month, and the DDM stock (*n*-dodecyl-β-d-maltoside [GoldBio, DDM5]) was prepared at 20% (w/v) in cold water and protected from light and kept at 4°C.

For selective permeabilization and fractionation, pellets were first resuspended in cytoplasmic lysis buffer containing 0.015% digitonin by pipetting (20 strokes with a 200 µL pipette) and incubated on ice for 10 min. Samples were centrifuged at 2000*g* for 5 min at 4°C, and the supernatant was collected as the cytosolic fraction, with small aliquots reserved for western blot analysis. Remaining supernatant was removed from the pellet, which was then gently washed (not resuspended) in cytoplasmic wash buffer (0.004% digitonin with the same buffer components as above) and immediately centrifuged at 2000*g* for 5 min at 4°C. The resulting supernatant was discarded.

Pellets were subsequently further lysed with ER lysis buffer containing 2% DDM by pipetting 10 times and incubating on ice for 10 min. Samples were centrifuged at 2400*g* for 5 min at 4°C, and the supernatant was collected as the ER-enriched membrane fraction, with small aliquots reserved for western blot analysis.

### Isolation of polysomes

HEK293 cells at 70%–80% confluency were incubated with 100 μg mL^−1^ cycloheximide for 3 min and collected by scraping in PBS. Cells were lysed for 10 min at 4°C in lysis buffer containing 20 mM Tris–HCl, 150 mM KCl, 5 mM MgCl_2_, 0.5% NP-40, 2 mM DTT, 100 μg mL^−1^ cycloheximide, protease inhibitor cocktail (cOmplete EDTA-free, Roche), and murine RNase inhibitor (NEB). Lysates were cleared by centrifugation at 12,000*g* for 15 min at 4°C. Cleared lysates were normalized based on NanoDrop UV spectrophotometer measurements (Thermo Fisher) and loaded onto 7%–47% (w/v) linear sucrose gradients (Sigma BioUltra) prepared in polysome buffer. Gradients were centrifuged at 35,000*g* for 3 h at 4°C using an SW 40 Ti rotor (Beckman). Fractions (1 mL) were collected from the top while continuously monitoring absorbance at 260 nm using the BioLogic LP system (Bio-Rad). Absorbance profiles were plotted to generate polysome profiles. Fractions corresponding to polysomes were pooled for total RNA isolation and RiboMeth-seq.

### Isolation of RNA

Total RNA isolation was performed using QIAZOL (Qiagen) and chloroform following the manufacturer's protocol. RNA concentrations were measured using a NanoDrop One spectrophotometer (Thermo Scientific) and RNA quality was assessed using an Agilent 2100 Bioanalyzer system with the Agilent RNA 6000 Nano Kit, according to the manufacturer's instructions.

### RiboMeth-seq

RiboMeth-seq library construction and sequencing were performed essentially as previously described (Birkedal et al. 2015; Krogh et al. 2016), except that libraries were pooled after 3′-adapter ligation containing sample-specific barcodes. Triplicate libraries were generated for each cell line or condition. A total of 1.5–4 µg of total RNA was used as input per library. RNA was partially degraded by alkaline hydrolysis at denaturing temperatures, and 20–40 nt fragments were purified on 10% TBE–urea polyacrylamide gels. 3′ adapters were ligated using a system based on a modified Arabidopsis tRNA ligase that joins 2′,3′-cyclic phosphate and 5′-phosphate termini. After 3′-adapter ligation with distinct barcodes for each sample, barcoded RNA fragments were pooled, and 5′-adapter ligation was performed similarly using the modified Arabidopsis tRNA ligase. The resulting libraries were processed on the Ion Chef system and sequenced using the Ion 540 Kit-Chef (Ion Torrent A30011) and Ion 540 Chip Kit (Ion Torrent A27766).

### RiboMeth-seq data analysis

Data were analyzed as previously reported (Birkedal et al. 2015; Krogh et al. 2016). Briefly, sequencing reads were mapped to a corrected human rRNA reference sequence. The RiboMeth-seq (RMS) score represents the fraction of molecules methylated at each nucleotide position and is calculated by comparing the number of read-end counts at the queried position to those at six flanking positions on either side. Quantification in human rRNA was performed for 41 sites in 18S, 67 in 28S, and two in 5.8S rRNA, for which both RMS and mass-spectrometry evidence are available. Statistically significant differences in RMS signatures between two cell lines or conditions were determined by pairwise comparison (*P* < 0.05, two-tailed unpaired Welch's *t*-test) combined with a minimum absolute change of 0.15 in RMS score. Dumbbell and heat map representations were generated in R using the ggplot2 and pheatmap packages. RMS data have been deposited in the Gene Expression Omnibus (GEO) under accession GSE325946.

The human 28S rRNA is encoded by multiple genomic copies that show sequence heterogeneity, leading to the coexistence of distinct 28S rRNA variants within a single cell. As a result, different numbering schemes have been used to annotate 2′-O-methylated residues in 28S rRNA. Earlier studies typically followed the 28S rRNA coordinates implemented in snoRNABase, whereas more recent work uses the NCBI RefSeq curated human 28S rRNA sequence NR_003287.4 (Bergeron et al. 2023), which we follow here. A correspondence table aligning snoRNABase and NR_003287.4 coordinates is provided in Supplemental Table S1; an equivalent conversion resource is also available in snoDB.

### Western blotting

Three percent of each cytoplasmic lysate, cytoplasmic wash, and ER fraction was separated by sodium dodecyl sulfate–polyacrylamide gel electrophoresis (SDS–PAGE) and transferred to PVDF membranes. The following primary antibodies were used: rabbit anti-Calnexin (Cell Signaling Technology 2679S; 1:1000), mouse anti-GAPDH (Santa Cruz Biotechnology sc-47724; 1:2000), rabbit anti-Histone H3 (Cell Signaling Technology 9756; 1:1000), rabbit anti-RPS6 (Cell Signaling Technology 2217; 1:4000), rabbit anti-RPL10A (RayBiotech 144-05925; 1:1000), mouse anti-Tuj1 (STEMCELL Technologies 60092; 1:500), and mouse anti-RPL22 (Santa Cruz Biotechnology sc-136413; 1:1000). Western blots were quantified using the open-source software ImageJ/Fiji. The sum of signals from the cytosol and ER fractions was set as 100%, and relative signals were calculated accordingly.

### RT-qPCR

For validation of cytosolic and ER RNA partitioning, total RNA was isolated separately from cytosolic and ER fractions, and an equal amount of RNA from each fraction was reverse-transcribed using MultiScribe Reverse Transcriptase (Invitrogen 4311235) to generate cDNA. For RT-qPCR assays, we used the same isolated RNA pool as for RiboMeth-seq whenever sufficient material remained after library preparation, and RT-qPCR was therefore performed only on samples with spare RNA, resulting in three biological replicates for HEK293 cells, two for neurons, and one for NPCs. Relative transcript abundance was assessed by quantitative PCR (qPCR) for the cytosolic markers *GAPDH* and *POLR2I* and ribosomal proteins *RPL12*, *RPL8*, *RPLP2*, and *RPS2*, as well as the ER chaperone *HSP90B1*, *PSAP*, and *COL3A1* (for qPCR primers, see Table 1). Relative fold changes between fractions were calculated for each marker using the ΔCt method, and the percentage contribution of cytosolic versus ER fractions was derived from the normalized relative expression values, taking into account the total RNA recovered from each fraction.

**TABLE 1.** Primers used in this study

Primer	Sequence (5′→3′)
GAPDH forward	AACGGATTTGGTCGTATTGG
GAPDH reverse	CTTCCCGTTCTCAGCCTTG
HSP90B1 forward	TGGGACTGGGAACTTATGAATG
HSP90B1 reverse	GGAGCAGATGTGGGTACAAATA
RPL12 forward	GCCTGAGGATTACAGTGAAACT
RPL12 reverse	GGTTCCTTGAGGGCTTTGAT
RPS2 forward	CTGGTATCGATGACTGCTACAC
RPS2 reverse	CTGATAGGGAGACTTGGTGAATAC
PSAP forward	GCATGGCCGACATATGCAAG
PSAP reverse	AACCAGCGCACAGATCTCC
COL3A1 forward	CACTGGTGGACAGATTCTAGTG
COL3A1 reverse	CTGGAGAGAAGTCGAAGGAATG
RPLP2 forward	CAGCGCCAAGGACATCAAGA
RPLP2 reverse	CAGCAGGTACACTGGCAAGC
POLR2I forward	GAGAACCGCATTCTGCTCTACG
POLR2I reverse	GTCGGCGATAATCTGGGTCAGT
RPL8 forward	CGGGATCCGTATCGGTTTAAG
RPL8 reverse	TCTCAGGGTTGTGGGAGATA

### Immunofluorescence

For immunofluorescence, cells were either grown and stained in LabTek chambers (Sigma-Aldrich C7182) or cultured on glass coverslips in 24 well plates. Cells were briefly washed with cold PBS without calcium and magnesium and fixed in 4% formaldehyde for 15 min at room temperature, followed by three washes in 1× PBS. Permeabilization and blocking were performed for 1 h at room temperature in 1× PBS containing 0.5% Triton X-100 and 5% FBS. Primary antibodies were diluted in the same buffer and incubated overnight at 4°C, then washed off with three 10 min washes in PBS. Secondary antibodies were diluted 1:1000 in blocking buffer and applied for 1 h at room temperature, followed by three additional 10 min PBS washes. Slides were mounted in Duolink In Situ mounting medium with DAPI (Sigma-Aldrich, DUO82020). Images were acquired on a Zeiss Axio Imager.M2 microscope (490020-0004-000) and analyzed using the open-source ImageJ software (Fiji).

The following primary antibodies were used: mouse anti-OCT4 (STEMCELL Technologies 60093; 1:1000), rabbit anti-PAX6 (STEMCELL Technologies 60094; 1:500), rabbit anti-Calnexin (Cell Signaling Technology 2679S; 1:50), and mouse anti-TUJ1 (STEMCELL Technologies 60092; 1:500).

## SUPPLEMENTAL MATERIAL

Supplemental material is available for this article.

## Figures and Tables

**Ülkü Uzun:**
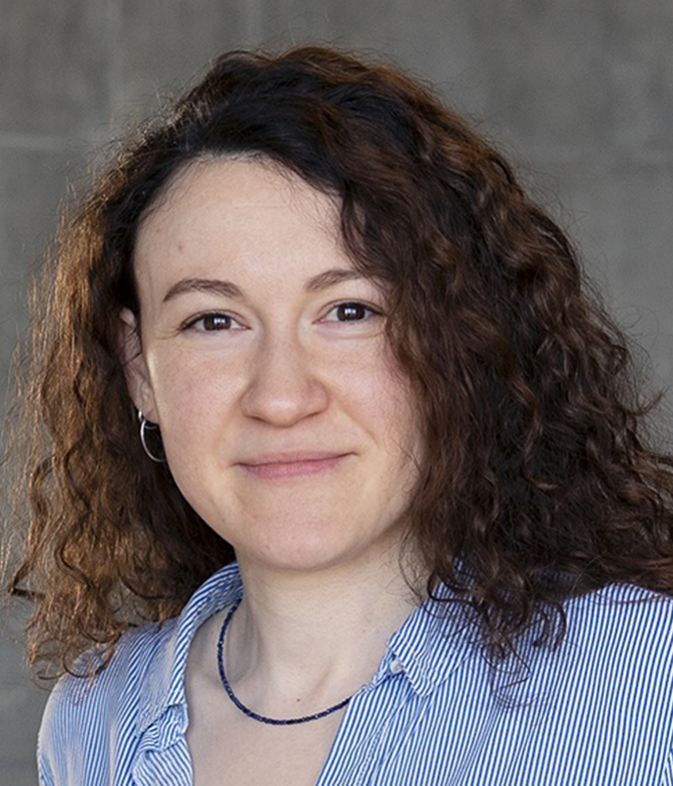


## MEET THE FIRST AUTHOR

***Meet the First Author(s)* is an editorial feature within *RNA*, in which the first author(s) of research-based papers in each issue have the opportunity to introduce themselves and their work to readers of *RNA* and the RNA research community. Ülkü Uzun is the first author of this paper, “Cytosolic and ER-associated ribosomes share rRNA 2′-O-methylation landscapes across human cell types.” Ülkü is an Assistant Professor at the Lund Lab, BRIC, University of Copenhagen. Her research focuses on RNA biology, with a particular emphasis on RNA localization and local translation, aiming to understand how spatial control of RNA contributes to gene expression regulation within cells.**

**What are the major results described in your paper and how do they impact this branch of the field?**

We show that cytosolic and ER-associated ribosomes share nearly identical rRNA 2′-O-methylation landscapes across HEK293 cells, neural progenitor cells, and neurons, with only minor site-specific differences. Instead, variation in 2′-O-Me is primarily driven by cell type rather than subcellular localization, indicating that this modification does not contribute to a general “ER vs. cytosol” ribosome identity. These findings point toward additional mechanisms—such as mRNA localization, ribosome-associated factors, and local biochemical environments—as key drivers of ER-specific translation, while not excluding that 2′-O-methylation may still play roles in more specialized or localized subcellular contexts.

**What led you to study RNA or this aspect of RNA science?**

I became interested in this area because I wanted to understand gene expression beyond transcription, especially how translation can be tuned by the ribosome itself. While it is well established that translation can be locally regulated within cells, whether ribosome heterogeneity contributes to this spatial control remains largely unknown. This gap motivated me to ask whether distinct ribosome populations might be selectively distributed across subcellular compartments, so we focused on ER-bound versus cytosolic ribosomes as defined translation environments.

**What are some of the landmark moments that provoked your interest in science or your development as a scientist?**

One key moment was when I first learned about Mendel and his pea plant experiments. I was fascinated that such a simple, curiosity-driven study could reveal fundamental rules of biology, giving me the sense that science is about asking the right questions and uncovering structure in what initially appears to be randomness. That realization stayed with me and was the first time I felt that I wanted to be part of such a creative and important process myself.

**What are your subsequent near- or long-term career plans?**

In the near term, I plan to continue in academia and establish my own research group focused on local translation. There is still limited understanding of how gene expression is controlled across distinct subcellular environments, despite its fundamental importance. I aim to uncover the mechanisms by which local protein synthesis is coordinated through the interplay between ribosomes, mRNA localization, and RNA-binding proteins. Ultimately, I hope to deepen our understanding of spatial gene expression and how its dysregulation contributes to human disease.
